# Economic evaluations of tobacco control mass media campaigns: a systematic review

**DOI:** 10.1136/tobaccocontrol-2014-051579

**Published:** 2014-07-01

**Authors:** Edwinah Atusingwize, Sarah Lewis, Tessa Langley

**Affiliations:** Division of Epidemiology and Public Health, Nottingham City Hospital, University of Nottingham, Nottingham, UK

**Keywords:** Economics, Media, Cessation

## Abstract

**Background:**

International evidence shows that mass media campaigns are effective tobacco control interventions. However, they require substantial investment; a key question is whether their costs are justified by their benefits. The aim of this study was to systematically and comprehensively review economic evaluations of tobacco control mass media campaigns.

**Methods:**

An electronic search of databases and grey literature was conducted to identify all published economic evaluations of tobacco control mass media campaigns. The authors reviewed studies independently and assessed the quality of studies using the Drummond 10-point checklist. A narrative synthesis was used to summarise the key features and quality of the identified studies.

**Results:**

10 studies met the inclusion criteria and were included in the review. All the studies included a cost effectiveness analysis, a cost utility analysis or both. The methods were highly heterogeneous, particularly in terms of the types of costs included. On the whole, studies were well conducted, but the interventions were often poorly described in terms of campaign content and intensity, and cost information was frequently inadequate. All studies concluded that tobacco control mass media campaigns are a cost effective public health intervention.

**Conclusions:**

The evidence on the cost effectiveness of tobacco control mass media campaigns is limited, but of acceptable quality and consistently suggests that they offer good value for money.

## Background

Tobacco use kills nearly six million people annually, and it is estimated that it will cause a billion deaths during the 21st century.^1^ International evidence has shown that tobacco control mass media campaigns (MMCs) can increase smoking cessation and reduce smoking prevalence and uptake in adults.^2–4^ There is also evidence that mass media can prevent the uptake of smoking in young people, although it is less robust.^5^ Campaigns tend to convey messages about the negative health consequences of smoking or more positive messages, such as information about how to quit smoking. Some campaigns have also shown tobacco industry marketing tactics.^6^ ^7^ Commonly used media channels for tobacco control MMCs include television, the internet, radio, billboards and print media.^8^

MMCs are able to deliver specific messages to large numbers of people; however, they require substantial upfront expenditures.^2^ ^8^ ^9^ For example, developing a new advertisement in Australia in 2010 from initial research including concept development and production required a minimum of 6 months and on average cost $A400 000.^10^ Existing guidelines recommend that governments in developed countries should spend about US$1.50–4.00 per person per year (approximately 15%–20% of total tobacco control expenditures) on antitobacco counter advertisements and health communication.^8^ In England, up to £38 million were spent on tobacco control marketing in 2009–2010.^11^

Given the costs associated with developing and implementing MMCs, it is particularly important to establish whether the public health benefits of such campaigns are sufficient to justify these expenditures. Economic evaluation involves the identification, measurement and valuation of costs and benefits of health interventions to establish whether they offer value for money. To our knowledge, there is currently no peer-reviewed systematic review of economic evaluations of tobacco control MMCs. This study aims to identify and critically assess published economic evaluations of tobacco control MMCs to establish whether they can be a worthwhile tobacco control intervention.

## Methods

This review was conducted according to the Centre for Reviews and Dissemination (CRD) guidance for undertaking systematic reviews of economic evaluations.^12^

### Search strategy

A search strategy was developed to identify all published economic evaluations of tobacco control MMCs. The following electronic databases were searched to identify relevant studies from inception to 15 May 2013: Medline, the Cochrane Library, Embase, PubMed, Web of Knowledge, the Health Economic Evaluations Database, the Cost Effectiveness Analysis registry (CEA) and the National Health Service Economic Evaluation Database. The literature search was rerun on 16 April 2014 to identify any relevant studies published since the original search date.

The search terms were developed in relation to the intervention, outcomes and designs of the studies. The search strategy for Medline, Web of Knowledge and Embase included both MeSH terms and free texts of the primary search terms. The search terms were:

- Mass media
- Campaign
- Advert*
- Marketing
- Promot*
- Social media
- Television
- Radio
- Billboard
- Tobacco
- Tobacco control
- Cigarette*
- Smok*
- Tobacco use
- Prevention
- Reduction
- Cessation
- Quit
- Economic evaluation
- Cost effectiveness
- Cost utility analysis
- Cost benefit analysis
- Cost effectiveness ratio

The reference lists of retrieved articles were also searched to identify potentially relevant studies. In addition, other online grey literature was identified using Google and Google Scholar. The website http://www.theses.com/ was also searched for relevant studies. Published articles without full texts online, but available in the University of Nottingham Library, were also considered.

### Inclusion criteria

This study used broad inclusion criteria to ensure that all economic evaluations of tobacco control MMCs would be included. Studies considered suitable for inclusion were those which evaluated the costs and benefits of MMCs.

#### Study design

We included studies which used standard economic evaluation designs such as CEA, cost utility analysis (CUA) or cost benefit analysis (CBA), as defined below:

- CEA: Benefits are measured in natural units (eg, life years gained (LYG), smokers averted) to obtain an incremental cost per outcome (eg, cost per additional quitter).
- CUA: Benefits are measured using a measure of utility (quality-adjusted life years (QALYs) or disability-adjusted life years (DALYs)) to obtain an incremental cost per QALY gained/DALY averted.
- CBA: Benefits are converted to monetary units to be compared with costs, deriving a cost benefit ratio.

In both CEA and CUA, the main result is usually expressed as an incremental cost effectiveness ratio (ICER)—the ratio of the change in costs to incremental benefits of an intervention. Policymakers can use this to help them decide if an intervention is an efficient use of resources, by making a judgment about the maximally acceptable cost per unit of outcome. There is usually no explicit ICER threshold, although implicit cost per QALY thresholds has been estimated from funding allocation decisions, for example, Australia (AU$69 900/QALY), New Zealand (NZ$20 000/QALY) and Canada (acceptance up to CAN$80 000/QALY, rejection from CAN$31 000 to CAN$137 000/QALY).^13^

#### Intervention

A tobacco control MMC was defined as using any of the following channels of communication to deliver a tobacco control message to a large population: television, radio, newspapers, billboards, internet, leaflets or booklets. The purpose of the MMC had to be to reduce the harms caused by tobacco consumption, for example, by encouraging cessation, reducing uptake or reducing secondhand smoke exposure. The MMC could be evaluated as a stand-alone intervention or as a part of a wider tobacco control programme. All target populations were considered.

#### Outcomes

All health and smoking-related outcomes were included; it was anticipated that the majority of studies would measure quit attempts, quitters, smokers averted, LYG (a measure of the additional number of years lived as a result of an intervention), QALYs (a measure of the additional number of years lived as a result of an intervention adjusted for quality of life in those years) or DALYs (years of life adjusted for the effect of illness on disability). Studies which measured the cost per person to see an advert were excluded.

#### Perspective

Studies were included regardless of the perspective of the evaluation, that is, they could take a narrow perspective, such as the National Health Service, or a broad societal perspective.

### Identification of papers

The lead reviewer (EA) conducted the literature search to identify relevant studies. Two reviewers screened the title and abstracts of the returned citations and considered them for inclusion using the criteria above. Full texts of potentially relevant articles were retrieved and independently screened by both reviewers to determine whether a study should be included. Disagreements were resolved through discussion and reasons for exclusion recorded.

### Data extraction and quality assessment

Data extraction and quality assessment were conducted by the lead reviewer and checked by a second reviewer.

Data extraction focused on key methodological features (type of economic evaluation, analytical approach (real data vs hypothetical modelling), outcome measures, study perspective, collection of cost data, time horizon, discounting, sensitivity analyses) and the nature of the intervention (setting, target population, type of mass media, duration, main campaign message). Background characteristics such as authors and years of study and publication were also extracted.

Quality assessment was conducted using the most widely used checklist for assessing the quality of economic evaluations, the BMJ checklist.^12^ ^14^ Although there are scoring systems for assessing the quality of economic evaluations, existing evidence suggests that they are not sufficiently valid and reliable, and the CRD guidelines recommend these are not used.^12^

Due to substantial heterogeneity between the studies, a meta-analysis was not possible. A narrative synthesis of the identified studies was used to summarise the key features of the identified studies, and compare study question, interventions, methods and results. We present descriptive critical assessment based on the BMJ checklist and summarise the quality of the studies using the Drummond 10-point checklist.^15^ This checklist covers the same main points as the BMJ checklist but is a quicker tool for assessing the quality of studies.

## Results

The electronic search in May 2013 identified 842 studies. One further study was identified by hand search of the reference lists of the included studies, making a total of 843 potential studies. Screening by title reduced this to 65. Abstract screening reduced this further to 22 potential studies, which were retrieved for full text review. Following full text review, a further 12 studies were excluded, leaving 10 studies to be included in the review.^16–25^ One of these studies was a report published by NICE; all others were studies published in peer-reviewed journals.^20^ A further relevant study in a peer reviewed journal was identified in the updated literature search.^26^ A flow diagram of the selection of studies and a list of studies excluded at the full text review stage, including reasons for exclusion, is provided in online supplementary appendices 1–3.

### Characteristics of studies

Table 1 reports the key characteristics of the included studies. Six studies were CEA,^16–19^ ^24^ ^26^ three were CUA,^20^ ^21^ ^23^ and two included both a CEA and a CUA.^20^ ^22^ All but two of the seven most recent studies included a CUA.

**Table 1:** Data extraction

Author, year, country	Population	Intervention/comparator	Effectiveness data	Measure(s) of benefit	Cost data	Reported perspective/discounting/time horizon	Sensitivity analysis	Results
Hurley and Matthews, 2008, Australia^22^	General population	National Tobacco Campaign, June–November 1997, graphic antismoking television advertisements/no intervention	Effectiveness of NTC on prevalence estimated from survey data. Markov model used to estimate future health benefits	LYG, QALYs	Cost of campaign adjusted to $A10.1 in 2001 $A. Healthcare cost estimated from existing literature, Future savings estimated using Markov model (lung cancer, AMI, stroke, COPD only)	Healthcare/3%/lifetime	Assume only half of reduction in smoking prevalence observed attributable to campaign	Prevention of 55 000 deaths, gains of 323 000 Lys, 407 000 QALYs, healthcare cost savings $A740.6 m. Campaign remained cost saving in sensitivity analysis
Higashi *et al*, 2011, Vietnam^23^	General population	Hypothetical MMCs implemented over 5 years (TV, radio, newspaper, journal, internet and electronic billboards)/no intervention	Effect of campaigns on uptake and cessation estimated from literature. Markov model used to estimate future health benefits	DALYs	Costed using WHO CostIt programme, 2006 VND: human resources requirements, media and advocacy, overheads. Healthcare cost savings for IHD, COPD and lung cancer	Government/3%/lifetime	ICER with and without healthcare cost offset	Without cost offset: VND 78 300 per DALY averted (95% CI 437 000 to 176 300). With cost offset: Campaign dominates
Kotz *et al*, 2011, UK^24^	General population	NSD, 1 day/year since 1984—national campaign aiming to create supportive environment and highlight available help for people who want to quit. National social marketing campaign/no intervention	Effect of NSD estimated from monthly survey data on quit attempts. Previously published model of cost effectiveness for smoking interventions used to estimate permanent rate of cessation and LYG	LYG	Estimated from NSD charity, report and financial statements—approximately £750 000, price year not stated	Organisational (NSD charity)/3.5%/lifetime	Assume that the true rate of permanent cessation attributable to NSD was only half that observed	ICER £82.24 per LYG (95% CI 49.7 to 231.6) for 35–44-year-olds. £114.29, £76.19 and £97.45 for age groups <35 years, 45–54 years and 55–64 years, respectively. Campaign remained cost effective in sensitivity analysis
Brown *et al*, 2014, UK^26^	General population	Stoptober—a 1-month national campaign in 2012 which set smokers the goal of being smoke-free for October	Effect of Stoptober estimated from monthly survey data on quit attempts. Previously published model of cost effectiveness for smoking interventions used to estimate permanent rate of cessation and LYG	LYG	Known costs of campaign obtained from Department of Health (2012 costs)	Organisational (Department of Health)/3.5%/lifetime	Examined effects of modelling different adjustments for relapse	ICER for total population £558 per LYG (95% CI 126 to 989). £414 for 35–44-year-olds, £607, 417 and 566 for <35-year-olds, 45–54-year-olds and 55–64-year-olds, respectively. Campaign remained cost effective in sensitivity analysis
Ratcliffe *et al*, 1997, Scotland^16^	Adults	Campaign aiming to reduce smoking prevalence via TV, posters and press advertising, a telephone helpline and a booklet containing cessation advice, October 1992–October 1993/no intervention	1-year cessation rate assessed by survey of helpline callers. Modelling used to estimate LYG	LYG	Retrospective analysis of costs of: development and maintenance, mass media advertising, telephone helpline, information booklet, costs. Costs attributable to adults only. Mass media represented two thirds of total cost. Price year not stated	Organisational/6% benefits/lifetime	Variation of campaign costs and number of helpline callers	Cost per discounted LYG range from £304 to £656 when parameters are varied
Villanti *et al*, 2012, USA^25^	Adults	EX campaign—television and radio campaign designed to promote smoking cessation, March–September 2008/no intervention	Survey used to estimate probabilities of confirmed awareness and quit attempts among those aware and those unaware of the EX campaign. National survey data used to estimate probability of quit attempts with no intervention. Probability of successful quitting from existing literature. Number of QALYs gained per quit from existing literature	QALYs	Intervention costs: Media, public relations, staff salaries. Other societal costs: smoking cessation medication, behavioural therapy. Medical treatment costs saved by quitting smoking assumed to be $0. Price year 2009	Societal/3%/lifetime	Variation of model parameters	Base case ICER $37 355. Sensitivity analysis: 95% uncertainty interval $10 779–204 976 per QALY
Fishman *et al*, 2005, USA^19^	18-year-olds in USA	Hypothetical 4-year MMC featuring regional and culturally relevant messages, using media outlets likely to reach adolescents/no intervention	Years of potential life saved among cohort of 18-year-olds based on existing literature	LYG	A range of assumed campaign costs based on existing literature—$0.31/head, $0.97/head, $2.35/head. Tobacco-attributable health costs from existing literature. Price year 2000	Societal/3%–7%/lifetime	Varying assumptions of campaign cost and discount rate	Cost per year potential life saved: $528 for low-cost media campaign with 3% DR, $19 957 for highest cost campaign with 7% DR
Secker-Walker *et al*, 1997, USA^17^	15–18-year-old students in four US cities	4-year TV and radio MMC in addition to school smoking prevention curriculum, 1986–1989. 36 TV ads and 17 radio ads specifically designed to appeal to students at different stages of adolescence/smoking prevention curriculum only	Difference in smoking prevalence between students in communities receiving intervention and those in comparator communities. Markov model used to estimate LYG	Smokers averted, LYG	Campaign development and production cost from campaign records, price year 1996. Air-time costs quoted by TV and radio stations. Costs estimated at community level and for whole of USA	Organisational/0%, 3%, 5%/lifetime	Different discount rates, different mass media effect sizes, different costs, halving LY lost due to smoking, variations in prevalence	Community level: cost per smoker averted $754 (95% CI 531 to 1296), cost per LYG at 3% DR $696 (95% CI 445 to 1269)National level: cost per smoker averted $162, cost per LYG at 3% DR $138 (95% CI 88 to 252) Campaign remained cost effective in sensitivity analysis
Raikou and McGuire, 2008, UK^20^	13–17-year-olds in the UK	Hypothetical 5-year MMC/no intervention	Effect on smoking prevalence estimated from the existing literature. Markov model used to estimate QALYs gained	QALYs, LYG	Campaign costs based on 10× cost of education and communication programmes used to support implementation of smoke-free legislation (price year not stated). Costs of treating smoking-related diseases from existing literature (2006 prices)	Public health sector/3.5%/lifetime	Varying assumptions of size of effect of intervention, cost of intervention and background quit rate	Base case: £49 per QALY gained £362 per LYG. Campaign remained cost effective in all sensitivity analyses
Holtgrave *et al*, 2009, USA^21^	12–17-year-olds in USA	National youth smoking prevention campaign (truth campaign), February 2000–2002. TV radio, online and print media elements, a campaign tour that followed youth music events/no intervention	Smokers averted estimated in previous study. QALYs gained estimated using data from existing literature	QALYs	Campaign cost data derived directly from expenditure records. Development and delivery of media elements, campaign tour, evaluation, litigation costs. Price year not stated, collected 2000–2002. Future healthcare costs saved estimated from existing literature (price year 2000)	Societal/3%/lifetime	Variation of smokers averted, QALYs gained per averted smoker, treatment costs saved	Base case: 178 290 QALYs gained. Cost-saving. Optimistic case: 1 050 000 QALYs, cost saving. Pessimistic case: $4302 per QALY
Stevens *et al*, 2002, UK^18^	Turkish community in Camden and Islington, London, UK	10 min play, poster campaign, leaflets. 1996–1997 (dates not specified)/no intervention	Before and after panel survey used to estimate effect of intervention on quitting. Estimates of 1-year quitters and LYG estimated from literature	1 year quitters, LYG	Actual expenditure from project records—salary costs, other labour costs, non-pay costs, overheads. Price year not stated, collected 1996–1997	Local authority/none/lifetime	Varying assumptions of population size, smoking population, quit rate, population smoking trend, continued abstinence, life years saved by quitting. Monte Carlo simulation	Study reports mean cost effectiveness drawn from probability distribution of possible outcomes in sensitivity analysis. ICER £105 per LYG (95% CI £33 to 391) ICER 825 per 1-year quitter (95% CI 300 to 3500)

$A, Australian dollar; AMI, acute myocardial infarction; COPD, Chronic Obstructive Pulmonary Disease; DALY, disability-adjusted life year; DR, discount rate; ICER, incremental cost effectiveness ratio; IHD, ischaemic heart disease; LY, life year; LYG, life years gained; MMC, mass media campaign; NSD, no smoking day; NTC, National Tobacco Campaign; QALY, quality-adjusted life year; VND, Vietnamese dollar.

There was wide variety between the included studies in terms of target population and type of MMC.

In four studies, the campaign targeted people under the age of 18;^17^ ^19–21^ in two, adults were targeted.^16^ ^25^ In four studies, the campaign was aimed at the general population.^22–24^ ^26^ One targeted a specific demographic group—the Turkish community in London.^18^

Eight studies were based on real-life MMCs and extrapolated the outcomes of those campaigns to obtain long-term cost effectiveness^16–18^ ^22^ ^24^ ^26^ or cost utility estimates.^21^ ^22^ ^25^ Three studies used data on the impact of MMCs to model the impact of hypothetical campaigns.^19^ ^20^ ^23^

All the MMCs aimed to reduce smoking by preventing uptake or encouraging cessation. No studies reported that the campaigns aimed to reduce exposure to secondhand smoke. The types and number of media channels used varied between studies, although all except one reported the use of television advertising.^20^ In other studies, radio,^17^ ^21^ ^23^ ^25^ ^26^ a range of print media^16^ ^21^ ^23^ ^26^ and internet-based media were used.^21^ ^23^ ^26^ One study reported the impact of a campaign which included a free telephone helpline and therefore did not just look at the effect of mass media.^16^ Other studies considered effects of mass media in conjunction with other tobacco control interventions, but provided the results specific to the MMC component, which have been reported in this review.^19^ ^20^ ^23^ One study, based on a hypothetical campaign, did not report which media were assumed to have been used.^20^ The duration of exposure was not always specified, but varied from an annual 1-day campaign^24^ to multiple campaigns over a 5-year period.^23^

The perspective of the economic evaluations also varied substantially, from very narrow, such as that of the organisation running the campaign,^16–18^ ^24^ ^26^ which tended not to include future healthcare costs, to wider perspectives where future healthcare costs were taken into account.^19–23^ ^25^

All studies reported an incremental analysis and made favourable conclusions regarding the cost effectiveness of the MMCs. Some studies reported that MMCs were cost saving when future healthcare costs were taken into account;^21–23^ others that took into account further healthcare costs did not find that campaigns were cost saving.^19^ ^20^ ^25^ The ICERs varied substantially even between studies using the same measure of benefit; this can be explained by the substantial variations in types and intensities of campaigns, study perspectives and costing methods (explained further below). The highest base case ICER was $37 355/QALY (price year 2009).

The ICERs also varied substantially within studies, depending on the assumptions made. However, even when pessimistic assumptions were used in sensitivity analysis (ie, higher costs and/or less effectiveness), results generally suggested that the evaluated campaign was cost effective. For example, a study based on a campaign targeting adolescents found that using optimistic assumptions the campaign was cost saving; in the pessimistic case, the ICER was $4302/QALY.^21^

Overall, substantial methodological heterogeneity between the studies makes it difficult to compare estimates between studies. In particular, no pattern emerged which indicated that a particular type of campaign, for example, those targeted at young people, was more likely to be cost effective than others.

### Quality assessment

On the whole, studies were well conducted bearing in mind substantial challenges associated with establishing effectiveness in population-level public health interventions. As demonstrated in table 2, the majority of studies had a clear research question, with a clear (although not usually explicitly stated) comparator of ‘no intervention’, which was appropriate. One study compared an MMC in addition to the school smoking prevention curriculum with the school smoking prevention curriculum only, which was equivalent to comparing with the status quo.^17^ All the studies used a study design that was appropriate to the research question—CEA or CUA—with an incremental analysis and a sensible measure of benefit: quit attempts, smokers averted, LYG, QALYs or DALYs. Furthermore, all studies attempted to demonstrate the long-run cost effectiveness of campaigns by using a lifetime horizon and conducted sensitivity analyses to allow for uncertainty in included parameters.

**Table 2:** Quality assessment

The 10-item Drummond checklist Was a well-defined question posed in answerable form? Was a comprehensive description of the competing alternatives given (ie, can you tell who did what to whom, where and how often)? Was the effectiveness of the programme or services established? Were all the important and relevant costs and consequences for each alternative identified? Were costs and consequences measured accurately in appropriate physical units (eg, hours of nursing time, number of physician visits, lost work-days, gained life years)? Were the cost and consequences valued credibly? Were costs and consequences adjusted for differential timing? Was an incremental analysis of costs and consequences of alternatives performed? Was allowance made for uncertainty in the estimates of costs and consequences? Did the presentation and discussion of study results include all issues of concern to users?

Author, year	1	2	3	4	5	6	7	8	9	10
Raikou, 2008	×	×	×	0	0	×	✓	✓	✓	×
Fishman, 2005	✓	×	✓	0	0	0	✓	✓	✓	×
Ratcliffe, 1997	✓	✓	✓	✓	✓	✓	0	✓	✓	✓
Holtgrave, 2009	✓	×	✓	✓	✓	✓	✓	✓	✓	✓
Hurley, 2008	✓	✓	✓	×	0	0	✓	✓	✓	✓
Stevens, 2002	×	✓	✓	✓	✓	0	×	✓	✓	×
Secker-Walker, 1997	✓	✓	✓	✓	✓	✓	✓	✓	✓	✓
Kotz, 2011	✓	×	✓	0	0	0	✓	✓	✓	×
Villanti, 2012	✓	✓	✓	✓	✓	✓	✓	✓	✓	×
Higashi, 2011	✓	✓	×	✓	✓	✓	✓	✓	✓	✓
Brown, 2014	✓	×	✓	✓	0	0	✓	✓	✓	✓

Yes=✓.

No=×.

Not clear=0 (in cases where the information provided was not satisfactory, thus making it difficult for the reviewer to make a conclusion).

None of the studies which reported using a societal perspective took into account wider societal costs such as the cost of absenteeism and may therefore be better defined as a healthcare perspective, although the healthcare sector may not have funded the campaigns.^19^ ^21^ ^25^ One study included campaign costs and healthcare costs but was reported as taking an organisational perspective.^17^ Other studies included the same costs and defined the perspective as public health sector^20^ and government.^23^ Estimates of future healthcare costs used varied; some studies assumed that there would be future healthcare cost savings, whereas others assumed that these savings would be offset by ex-smokers living longer.

Several studies provided very limited detail about the MMCs being investigated.^19–21^ ^24^ Similarly, several studies had weaknesses in the reporting of costs. For example, one study reported an overall cost of a campaign with no breakdown of costs.^24^ Ideally, economic evaluations of MMCs should present detailed breakdowns of the elements involved in developing and running campaigns and the associated costs.

Estimating the effectiveness and long-run benefits of population-level public health interventions is known to be challenging. This is due to the nature of MMCs, most of which are implemented at the population level and which therefore cannot be easily evaluated in controlled trials. Of the included studies, eight out of 11 estimated the effectiveness of real life campaigns using primary survey data, or using existing estimates also based on surveys. These self-reported estimates of smoking behaviour are prone to under-recording, though comparisons of the same outcomes before and after a campaign are less likely to be affected. Findings are also potentially subject to confounding by other simultaneous interventions. All of the included studies extrapolated short-term effectiveness results over long-run to estimate LYG, DALYs or QALYs by making assumptions about key parameters, such as the underlying quit rate, what proportion of quit attempts will be successful in the long-run and sustained effects on prevention. This introduced further uncertainty into final results, but was necessary to estimate long-term the benefits of the campaigns.

Two of the included studies used monthly survey data on quit attempts collected over several years.^24^ ^26^ Although the monthly sample sizes were small, this enabled existing trends in quit attempts to be taken into account and robust comparisons to be made between periods with and without an MMC. These data were extrapolated to estimate long-term cessation rates using data from the existing literature. Similarly, in another study the authors extrapolated data from a cohort survey on self-reported campaign awareness and quit attempts to estimate long-term successful cessation.^25^ This is a standard approach, but introduces further uncertainty into the estimate of effectiveness. This uncertainty is reflected by the very wide uncertainty interval reported in the study.

Another study highlights the challenge of evaluating the effectiveness of a campaign in a small population with no regular data collection over time. In this study, effectiveness was assessed using a small panel survey with a low rate of follow-up.^18^ This approach seems reasonable given the setting, but introduces substantial uncertainty into the estimate of effectiveness: existing trends are not taken into account and it is difficult to determine a causal effect of the campaign. However, substantial sensitivity analysis in which several key parameters were varied did not change the study's conclusions.

Three of the included studies used estimates of campaign effects from the existing literature; while it is reasonable to assume based on existing literature that MMCs will be effective in both preventing uptake and increasing cessation, the specific estimates used are likely to be outdated^19^ ^23^ or not relevant to the study setting.^20^ ^23^ In two of the studies, a systematic review does not appear to have been carried out to determine the most suitable estimates to use.^20^ ^23^

All of the studies conducted sensitivity analyses, which was particularly important bearing in mind the limitations in the estimates of effectiveness and long-term benefits.

Most studies had a key element of benefits or costs that they were unable to take account of, and therefore the point estimates are unlikely to represent the true estimate. For example, one study estimated the benefits and cost savings for the cohort of 18-year-olds only; the authors acknowledge that their estimate is conservative given that MMCs also have an effect on adult smoking.^19^

## Discussion

This review found only 10 economic evaluations of tobacco control MMCs. Most of these were found to be of acceptable quality, although methodologies varied substantially. Some evaluated campaigns targeted adults, whereas others evaluated campaigns aimed at adolescents. All found the cost effectiveness profile of the evaluated campaign to be favourable.

The main strength of this review is that the broad search strategy ensured that all economic evaluations of MMCs, regardless of setting and target population, have been included. As a result, however, the types of campaigns and study methodologies were highly heterogeneous, making it difficult to compare studies and their results to draw definitive conclusions about which types of campaign are most cost effective. In particular, this heterogeneity precluded the use of meta-analysis. This is consistent with a previous review of studies on the cost effectiveness of health communication programmes, which identified considerable variety in methodologies and hence problems of transparency, comparability and generalisability.^27^

All of the included studies conducted incremental analyses and the majority of recent studies use CUA. In all the CUA, the interventions were either cost saving or the ICERs were well below commonly used thresholds, even in the case of models using pessimistic assumptions. The cost effectiveness estimates varied substantially, however. Aside from variations in the nature of the interventions and their direct costs, this seems likely to stem from variations in assumptions about the impact of interventions on future healthcare costs. These were, in all studies, taken from existing data sources, but were not based on systematic reviews. Some studies assumed that the interventions would save healthcare costs in the future; some assumed that they would be offset by the costs of quitters living longer. Different studies also accounted for healthcare costs for different diseases. For example, one study included only the healthcare cost savings for ischaemic heart disease, chronic obstructive pulmonary disease and lung cancer, and the cost savings may therefore have been underestimated.^22^

A further factor contributing to heterogeneity in the ICERs between the studies is the variety between the applied estimates of effectiveness. They are generally based on a single outcome, which varies and is measured differently between studies. None of these estimates provide a comprehensive assessment of campaign effectiveness, which could act on a diverse range of short and long outcomes, from awareness to prevalence.

The methods of establishing effectiveness in the included studies generally had some limitations; however, this is to be expected when studying the effects of population-level natural experiments, for which randomised controlled trials are not feasible. High quality population-level data on relevant outcomes are often not available, and therefore most studies rely on before and after surveys, making it difficult to take account of existing trends and confounding events. Regular cross-sectional surveys such as the Smoking Toolkit study (used in two of the included studies)^24^ ^26^ help to improve the evidence for the effectiveness of such interventions.

Of the three studies which modelled the cost effectiveness of hypothetical campaigns, two did not seem to use an estimate of effectiveness based on a systematic review. This is an element which could be improved through adherence to a recognised checklist for the reporting of economic evaluations such as the BMJ checklist or the more recent Consolidated Health Economic Evaluation Reporting Standards checklist.^14^ ^28^

All studies extrapolated campaign effectiveness estimates to obtain long-run estimates of life-years, QALYs or DALYs gained which inevitably introduced additional uncertainty into the findings; however, this is necessary due to the long lag to the onset of smoking-related morbidities. All studies reported sensitivity analyses and/or CIs which suggested that tobacco control campaigns are likely to be cost effective even if effectiveness is lower and/or costs higher than assumed in the base case.

Overall, the studies reviewed were of acceptable quality, but could have been improved in two key areas. First, the interventions were often poorly described in terms of campaign content and intensity, albeit that in some cases existing papers or campaign report may contain additional details. Second, cost information was frequently inadequate. Again, these elements could be improved through adherence to a recognised checklist for the reporting of economic evaluations.

Overall, the variety in methodology and varying level of quality are in line with that identified in the review of studies on the cost effectiveness of health communication programmes mentioned above.^27^ In addition, the evaluations were conducted in a limited range of countries—the UK, the USA and Australia—and the issue of generalisability was generally not addressed in the included studies. It is unclear whether the study findings can be transferred to other developed countries or middle income countries. In future research, it would be useful to explore the cost effectiveness of adapting existing campaigns for use in middle income countries, where the cost of developing and running new campaigns may be prohibitive.

Despite some common limitations in the literature, and methodological inconsistencies between studies, the evidence reviewed in this study consistently suggests that tobacco control MMC offers good value for money, with estimates well within commonly used thresholds for cost effectiveness. Given the nature of MMCs, this is perhaps unsurprising; they deliver targeted messages to large populations of people at a low cost per head. The evidence is highly limited, however, and there is scope for further studies which adhere to standard cost effectiveness methodologies and reporting guidelines, particularly outside of the UK and the USA.

**Key messages**

- Few studies on the cost-effectiveness of tobacco control mass media campaigns have been conducted.
- Existing studies are of acceptable quality and consistently suggest that such campaigns offer good value for money.

## Supplementary Material

Web supplement
