# Supplementary material for: Economic evaluations of tobacco control mass media campaigns: a systematic review
Source: Tob Control. 2014 Jul 1;24(4):320–7. doi: 10.1136/tobaccocontrol-2014-051579 (PMC4484382; doi:10.1136/tobaccocontrol-2014-051579)
Supplement: Web supplement [file tobaccocontrol-2014-051579-s1.pdf]

Appendix 1 – Search strategy

Search Strategy and Results: WEB OF KNOWLEDGE (All Databases), all years 1864 TO 2013, MAY

SET	RESULTS/HITS	COMBINATION (“OR” or “AND” TERMS)
# 8	**Approximately 1,375**	#7 AND #5 AND #4<br>*Timespan=All years*<br>*Search language=Auto*
# 7	**Approximately 131,968**	#2 AND #1<br>*Timespan=All years*<br>*Search language=Auto*
# 6	**Approximately 30,550**	#5 AND #4 AND #3<br>*Timespan=All years*<br>*Search language=Auto*
# 5	**Approximately 1,011,485**	Topic=(costeffectiveness) OR Topic=(Cost -effectiv*) OR Topic=(Economic Evaluation) OR Topic=(Cost benefit analysis) OR Topic=(CostUtility analysis) OR Topic=(Cost-effectiveness ratio)<br>*Timespan=All years*<br>*Search language=Auto*
# 4	**Approximately 21,329,215**	Topic=(tobacco) OR Topic=(Cigarette*) OR Topic=(Smok*) OR Topic=(Tobacco use) OR Topic=(Tobacco control) OR Topic=(control) OR Topic=(tobacco Prevention) OR Topic=(tobacco Reduction) OR Topic=(tobacco Cessation) OR Topic=(tobacco Quit*)<br>*Timespan=All years*<br>*Search language=Auto*
# 3	**Approximately 13,266,850**	#2 OR #1<br>*Timespan=All years*<br>*Search language=Auto*
# 2	**Approximately 1,179,350**	Topic=(Campaign*) OR Topic=(Mass media campaign*) OR Topic=(Advertising) OR Topic=(Advert*) OR Topic=(Marketing)<br>*Timespan=All years*<br>*Search language=Auto*
# 1	**Approximately 12,219,468**	Topic=(mass media) OR Topic=(Social media) OR Topic=(Media) OR Topic=(Television*) OR Topic=(Radio*) OR Topic=(Newspaper*) OR Topic=(Internet) OR Topic=(Billboard*)<br>*Timespan=All years*<br>*Search language=Auto*

Search Strategy and Results: MEDLINE (OVID)-1948-Present

#	Searches	Results	Search Type
1	Mass media.mp. or exp mass medium/	11093	
2	media.mp.	339172	
3	social media.mp. or exp social media/	1095	Advanced
4	Television*.mp. or exp television/	33093	Advanced
5	Radio*.mp.	1079442	Advanced
6	Newspaper*.mp.	11	Advanced
7	internet.mp. or exp Internet/	63599	Advanced
8	billboard*.mp.	234	Advanced
9	Campaign*.mp.	26504	Advanced
10	mass media campaign*.mp.	453	Advanced
11	Advertising.mp. or exp advertizing/	15179	Advanced
12	Advert*.mp.	18463	Advanced
13	exp marketing/ or Marketing.mp. or exp social marketing/	39835	Advanced
14	Promotion.mp.	91881	Advanced
15	1 or 2 or 3 or 4 or 5 or 6 or 7 or 8 or 9 or 10 or 11 or 12 or 13 or 14	1613346	Advanced
16	tobacco.mp. or exp tobacco/ or exp smokeless tobacco/ or exp tobacco industry/ or exp tobacco dependence/ or exp tobacco smoke/	83277	Advanced
17	Cigarette*.mp. or exp electronic cigarette/	48617	Advanced
18	exp smoking/ or Smok*.mp.	221449	Advanced
19	Tobacco-use.mp.	16581	Advanced
20	tobacco control.mp.	3801	Advanced
21	tobacco prevention.mp.	419	Advanced
22	tobacco reduction.mp.	52	Advanced
23	exp smoking cessation/ or tobacco cessation.mp.	20896	Advanced
24	tobacco quit*.mp.	109	Advanced
25	16 or 17 or 18 or 19 or 20 or 21 or 22 or 23 or 24	255920	Advanced
26	Cost effectiveness.mp. or exp "cost effectiveness analysis"/	32724	Advanced
27	Cost Effectiv*.mp.	70240	Advanced
28	Economic Evaluation.mp. or exp economic evaluation/	4770	Advanced
29	Cost benefit analysis.mp. or exp "cost benefit analysis"/	61271	Advanced
30	Cost utility analysis.mp. or exp "cost utility analysis"/	1116	Advanced
31	cost effectiveness ratio.mp.	3241	Advanced
32	26 or 27 or 28 or 29 or 30 or 31	105660	Advanced
33	15 and 25 and 32	420	Advanced
34	limit 33 to english language	400	Advanced

Search strategy and results: EMBASE- 1980 to 2013, May

#	Searches	Results	Search Type
1	Mass media.mp. or exp mass medium/	15726	Advanced
2	media.mp.	354547	Advanced
3	social media.mp. or exp social media/	1751	Advanced
4	Television.mp. or exp television/	18750	Advanced
5	Radio.mp.	36111	Advanced
6	News paper.mp.	21	Advanced
7	internet.mp. or exp Internet/	82923	Advanced
8	billboard.mp.	102	Advanced
9	Campaign.mp.	20154	Advanced
10	mass media campaign.mp.	219	Advanced
11	Advertising.mp. or exp advertizing/	18479	Advanced
12	Advert.mp.	90	Advanced
13	exp marketing/ or marketing.mp. or exp social marketing/	54760	Advanced
14	Promotion.mp.	115214	Advanced
15	1 or 2 or 3 or 4 or 5 or 6 or 7 or 8 or 9 or 10 or 11 or 12 or 13 or 14	670852	Advanced
16	tobacco.mp. or exp tobacco/ or exp smokeless tobacco/ or exp tobacco industry/ or exp tobacco dependence/ or exp tobacco smoke/	93917	Advanced
17	Cigarette.mp. or exp smoking/	224446	Advanced
18	Smok*.mp.	299488	Advanced
19	Tobacco-use.mp.	11235	Advanced
20	tobacco control.mp.	4094	Advanced
21	tobacco prevention.mp.	471	Advanced
22	tobacco reduction.mp.	51	Advanced
23	exp smoking cessation/ or tobacco cessation.mp.	35531	Advanced
24	tobacco quit.mp.	44	Advanced
25	16 or 17 or 18 or 19 or 20 or 21 or 22 or 23 or 24	336182	Advanced
26	Cost effectiveness.mp. or exp "cost effectiveness analysis"/	105745	Advanced
27	Cost Effectiv*.mp.	140218	Advanced

28	Economic Evaluation.mp. or exp economic evaluation/	203708	Advanced
29	Cost benefit analysis.mp. or exp "cost benefit analysis"/	66382	Advanced
30	Cost utility analysis.mp. or exp "cost utility analysis"/	5390	Advanced
31	cost effectiveness ratio.mp. or exp "cost effectiveness analysis"/	90255	Advanced
32	26 or 27 or 28 or 29 or 30 or 31	243394	Advanced
33	15 and 25 and 32	773	Advanced
34	limit 33 to english language	729	Advanced

Search strategy and results: PubMed

No	Searches	Result
**1**	((((cost effectiveness) AND tobacco) AND control) AND Mass media) AND Campaign*	11
**2**	((((cost effectiveness) AND tobacco) AND control) AND Mass media) AND Mass media campaign*	3
**3**	((((cost effectiveness) AND tobacco) AND control) AND Mass media) AND Advert*	7
**4**	((((cost effectiveness) AND tobacco) AND control) AND Mass media) AND Advertising	7
**5**	((((cost effectiveness) AND tobacco) AND control) AND Mass media) AND Marketing	5
**6**	((((cost effectiveness) AND tobacco) AND control) AND Mass media) AND Promot*	10
**7**	((((Cost effectiveness) AND tobacco) AND control) AND social media) AND Campaign	1
**8**	((((Cost effectiveness) AND tobacco) AND control) AND media) AND Campaign	10
**9**	((((Cost effectiveness) AND tobacco) AND control) AND Television*) AND Campaign	3
**10**	((((Cost effectiveness) AND tobacco) AND control) AND Radio*) AND Campaign	1
**11**	((((Cost effectiveness) AND tobacco) AND control) AND Internet) AND Campaign	0
**12**	((((Cost effectiveness) AND tobacco) AND control) AND billboard*) AND Campaign	1
**13**	((((Cost effectiveness) AND Cigaret*) AND control) AND Mass media) AND Campaign	1
**14**	((((Cost effectiveness) AND Cigarette) AND control) AND Mass media) AND Campaign	1
**15**	((((Cost effectiveness) AND Smok*) AND control) AND Mass media) AND Campaign	9
**16**	((((Cost effectiveness) AND Smoking) AND control) AND Mass media) AND Campaign	9
**17**	((((Cost effectiveness) AND Tobacco use) AND control) AND Mass media) AND Campaign	7

**18**	((((Cost effectiveness) AND Tobacco) AND Prevention) AND Mass media) AND Campaign	6
**19**	((((Cost effectiveness) AND Tobacco) AND Reduction) AND Mass media) AND Campaign	2
**20**	((((Cost effectiveness) AND Tobacco) AND Cessation) AND Mass media) AND Campaign	6
**21**	((((Cost effectiveness) AND Tobacco) AND Quit*) AND Mass media) AND Campaign	4
**22**	((((Cost -effectiv*) AND Tobacco) AND Control) AND Mass media) AND Campaign	6
**23**	((((Economic Evaluation) AND Tobacco) AND Control) AND Mass media) AND Campaign	12
**24**	((((Cost benefit analysis) AND Tobacco) AND Control) AND Mass media) AND Campaign	5
**25**	((((Cost-utility analysis) AND Tobacco) AND Control) AND Mass media) AND Campaign	0
	Total results	127

Appendix 2. PRISMA diagram of study selection

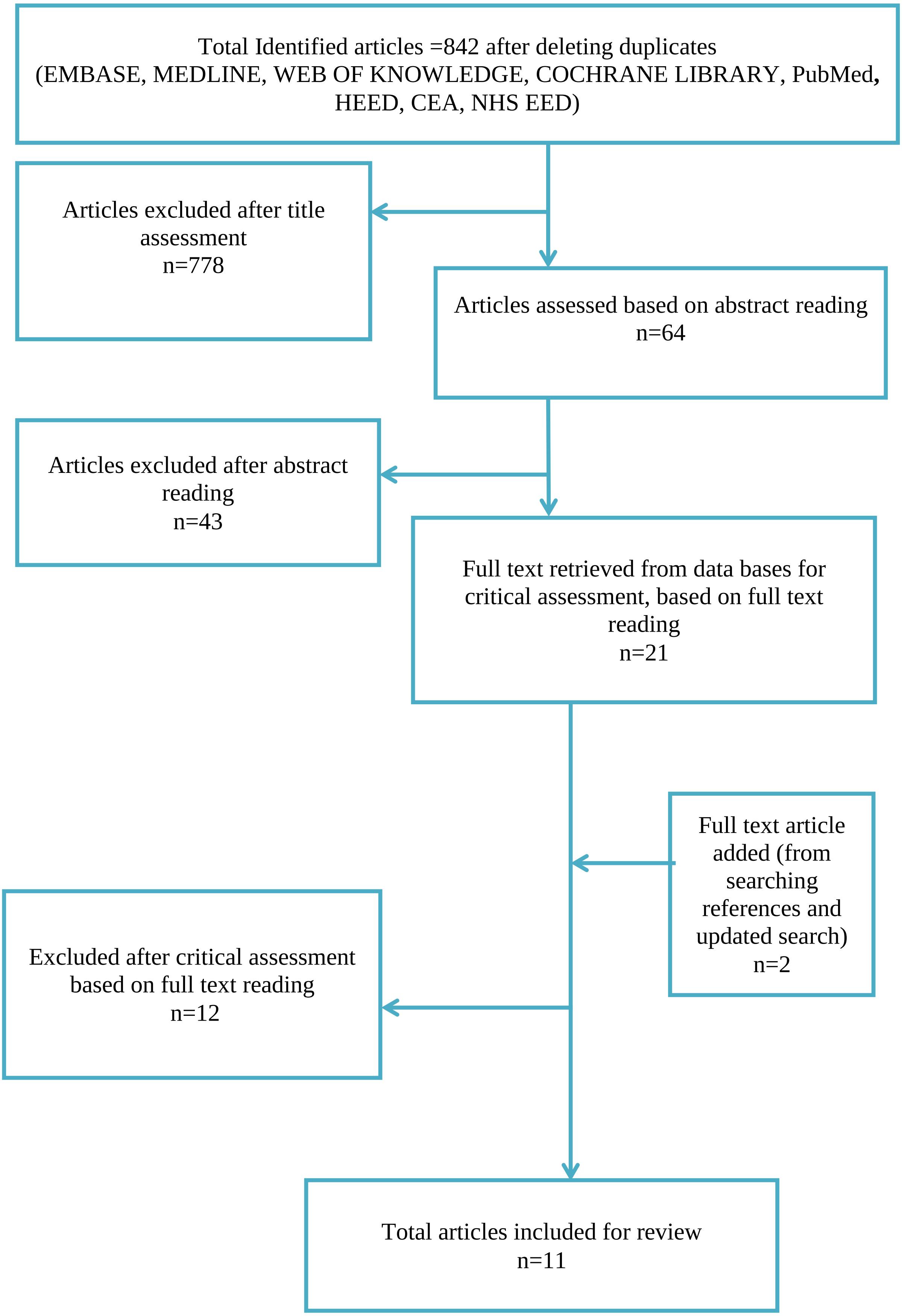

Appendix 3. Studies excluded after full text review

Study	Reason for exclusion
Fellows, J. L., Bush, T., McAfee, T. & Dickerson, J. Cost effectiveness of the Oregon quitline "free patch initiative". *Tobacco Control* **16 Suppl 1**, i47-52 (2007).	Not an economic evaluation of a mass media campaign (economic evaluation of adding a 2-week supply of free NRT for tobacco users registering for telephone counselling to generate calls to a quitline).
Tillgren, P. *et al.* Cost-effectiveness of a tobacco 'quit and win' contest in Sweden. *Health Policy* **26**, 43-53 (1993).	Not an economic evaluation of a mass media campaign (although the contest involved some mass media activities).
Farrelly, M. C., Hussin, A. & Bauer, U. E. Effectiveness and cost effectiveness of television, radio and print advertisements in promoting the New York smokers' quitline. *Tobacco Control* **16 Suppl 1**, i21-23 (2007).	Not a standard economic evaluation design (calculated increase in calls per $1000 spent on mass media).
Perl, R. *et al.* Mass media campaigns within reach: effective efforts with limited resources in Russia's capital city. *Tobacco Control* **20**, 439-441, doi:10.1136/tc.2010.041269 (2011).	Not an economic evaluation of a mass media campaign.
Bentz, C. J. *et al.* The feasibility of connecting physician offices to a state-level tobacco quit line. *American Journal of Preventive Medicine* **30**, 31-37 (2006).	Not an economic evaluation of a mass media campaign.
Hardigan, P. C., Succar, C. T. & Fleisher, J. M. An analysis of response rate and economic costs between mail and web-based surveys among practicing dentists: a randomized trial. *Journal of Community Health* **37**, 383-394,	Not an economic evaluation of a mass media campaign.
Parker, D. R. *et al.* Feasibility, cost, and cost-effectiveness of a telephone-based motivational intervention for underserved pregnant smokers. *Nicotine & Tobacco Research* **9**, 1043-1051 (2007).	Not an economic evaluation of a mass media campaign.
Chen, Y. F. *et al.* Effectiveness and cost-effectiveness of computer and other electronic aids for smoking cessation: a systematic review and network meta-analysis. *Health Technology Assessment (Winchester, England)* **16**, 1-205, iii-v, (2012).	Not an economic evaluation of a mass media campaign.
Smith, M. W., An, L. C., Fu, S. S., Nelson, D. B. & Joseph, A. M. Cost-effectiveness of an intensive telephone-based intervention for smoking cessation. *Journal of Telemedicine & Telecare* **17**, 437-440, (2011).	Not an economic evaluation of a mass media campaign.
Pechmann, C. & Reibling, E. T. Anti-smoking advertising campaigns targeting youth: case studies from USA and Canada. *Tobacco Control* **9**, 18-31 (2000).	Not an economic evaluation of a mass media campaign.
Modayil, M. V., Consolacion, T. B., Isler, J., Soria, S. & Stevens, C. Cost-effective smoke-free multiunit housing media campaigns: connecting with local communities. *Health promotion practice* **12** (2011).	Measured the cost per person to see an advert (i.e. not a health or smoking-related outcome)
Anonymous. Web programs can be helpful, cost-effective way to stop smoking. *Mayo Clinic Women's Healthsource* **13**, 3 (2009).	No full text available.
